# Phylogenetic Studies of *Coxiella*-Like Bacteria and Spotted Fever Group *Rickettsiae* in Ticks Collected From Vegetation in Chaiyaphum Province, Thailand

**DOI:** 10.3389/fvets.2022.849893

**Published:** 2022-04-06

**Authors:** Pawiga Usananan, Warissara Kaenkan, Ronnayuth Sudsangiem, Visut Baimai, Wachareeporn Trinachartvanit, Arunee Ahantarig

**Affiliations:** ^1^Biodiversity Research Cluster, Department of Biology, Faculty of Science, Mahidol University, Bangkok, Thailand; ^2^Center of Excellence for Vectors and Vector-Borne Diseases, Faculty of Science, Mahidol University, Nakhon Pathom, Thailand

**Keywords:** *Coxiella*-like bacteria, *Rickettsia*, *Haemaphysalis*, *Amblyomma*, tick

## Abstract

Ticks can transmit a wide variety of pathogens, including bacteria. Here, we report the detection of tick-associated bacteria in Chaiyaphum Province, northeastern Thailand. There have been few reports of tick-borne bacterial pathogens in the study areas, which are evergreen forests dominated by plateaus at elevations of approximately 1,000 m. In total, 94 ticks were collected from vegetation. They were screened for the presence of *Coxiella, Francisella, Rickettsia*, and *Borrelia* bacteria using PCR assays. In this study, we found ticks from two genera, *Haemaphysalis* and *Amblyomma*, that were positive for *Coxiella*-like bacteria (CLB) and *Rickettsia*. *Francisella* and *Borrelia* spp. were not detected in these two tick genera. The results revealed the evolutionary relationships of CLB in *Amblyomma testudinarium, Haemaphysalis lagrangei*, and *Haemaphysalis obesa* ticks using the *16S* rRNA and *rpoB* markers, which clustered together with known isolates of ticks from the same genera. In contrast, the *groEL* marker showed different results. On the basis of the *groEL* phylogenetic analysis and BLAST results, three groups of CLB were found: (1) CLB from *A. testudinarium* grouped as a sister clade to CLB from *Ixodes ricinus*; (2) CLB from *Haemaphysalis lagrangei* was distantly related to CLB from *Haemaphysalis wellingtoni*; and (3) CLB from *A. testudinarium* grouped as sister clade to CLB from *Amblyomma* from French Guiana and Brazil. For *Rickettsia* studies, phylogenetic trees of the *gltA, ompB*, and *sca4* genes revealed two groups of Spotted Fever Group (SFG) *Rickettsiae*: (1) SFG *Rickettsiae* that formed a sister clade with *Rickettsia tamurae* AT-1 (belong to the *Rickettsia helvetica* subgroup) in *A. testudinarium* and (2) SFG *Rickettsiae* that formed a distantly related group to *Rickettsia rhipicephali* 3-7-female6-CWPP (belong to the *Rickettsia massiliae* subgroup) in *A. testudinarium*. This study expanded our knowledge of the diversity of tick-borne *Coxiella* and *Rickettsia* bacteria. The pathogenic roles of these bacteria also need to be investigated further.

## Introduction

Ticks are important hematophagous ectoparasites of both humans and animals. They can transmit a wide range of pathogens, such as *Coxiella, Francisella, Borrelia*, and *Rickettsia* bacteria (1–4). Hard ticks, such as the genera *Amblyomma, Dermacentor*, and *Rhipicephalus*, have been identified as the main vectors of Spotted Fever Group (SFG) *Rickettsiae* (5). Several tick species have been reported in Chaiyaphum Province, northeastern Thailand. These include *Amblyomma testudinarium, Haemaphysalis asiatica, Haemaphysalis hystricis, Haemaphysalis semermis, Rhipicephalus haemaphysaloides*, and *Ixodes granulatus* (6). *Rickettsia* spp. are gram-negative bacteria that cause SFG diseases. SFG rickettsioses have been reported in many regions of the world, including Japan, northern China, Korea (7–9), and Thailand (10). SFG rickettsioses are characterized by fever, headache, muscle pain, maculopapular rash, and developing eschar at the site of tick bites (11). In Thailand, *Rickettsia* spp. have been reported near the Thai-Myanmar border, such as *Rickettsia* sp. strain RDla420 identified in *Dermacentor auratus* ticks obtained from a bear and *Rickettsia* sp. strain RDla440 detected in *Dermacentor* larval ticks from a wild pig nest (12). In addition, Sumrandee et al. (13) reported the first evidence of a *Rickettsia* sp. that is closely related to *Rickettsia tamurae* in *Rhipicephalus* (*Boophilus*) *microplus* ticks from Thailand. In Chaiyaphum Province, Malaisri et al. (14) reported a phylogenetic analysis of new *Rickettsia* genotypes that were closely related to *Rickettsia tamurae* and *Rickettsia monacensis* and might be pathogenic to humans.

Q fever is a zoonotic disease caused by *Coxiella burnetii*; infection mainly arises through the inhalation of airborne particles contaminated with bacteria. The clinical features of Q fever include flu-like symptoms to pneumonia and granulomatous hepatitis in serious cases (15). *Coxiella burnetii* infections and *Coxiella*-like bacteria (CLB) have been found in humans and other animals in Thailand (16–19). For example, *C. burnetii* was found in Thai patients in Khon Kaen Province, northeastern Thailand (20). CLB can promote the reproductive fitness and development of *Haemaphysalis longicornis* ticks (21). In addition, CLB can interfere with the colonization and transmission of pathogens. For example, CLB can impact pathogen susceptibility in ticks, e.g., CLB can defend their *Rhipicephalus haemaphysaloides* tick hosts against the pathogenic microbe *Babesia microti* (22). Interestingly, CLB was determined to be the cause of death in a female eclectus parrot (*Eclectus roratus*) (23). In Thailand, CLB was also detected in *Haemaphysalis* ticks, such as *Haemaphysalis shimoga* and *Haemaphysalis lagrangei* (24). Moreover, Trinachartvanit et al. (25) reported CLB in *Haemaphysalis wellingtoni* tick-infested fowl from various parts of Thailand.

The objectives of this study were to identify the presence of bacteria in ticks collected from vegetation and to reveal the overall diversity of bacterial infections and species in ticks using PCR and phylogenetic analysis.

## Materials and Methods

### Tick Collection and Identification

In 2014–2015, ticks (larvae, nymphs, and adults) were collected from vegetation from forests in Chaiyaphum Province, Thailand (16°16′25.2″N 101°29′02″E; 16°22′23.0″N 101°46′38.7″E; and 16°12′18.9″N 101°52′22.9″E). The areas being studied are evergreen forests dominated by plateaus at elevations of approximately 1,000 m. We placed the ticks into tubes that were held in a container with liquid nitrogen and stored them in a freezer after returning to the laboratory. Morphological identification of all tick stages was performed under a stereomicroscope (26, 27). Tick identification was performed using molecular methods on the ticks that were positive for either *Coxiella* or *Rickettsia* bacteria; previously published primers (16S+1/16S-1) for the amplification of mt *16S* rDNA were used (28). There were seven pools of *Haemaphysalis* ticks at the immature stage, including nymphs (four pools: three pools of five and one pool of four) and larvae (three pools: one pool of six, one pool of seven, and one pool of 15). For the *Amblyomma* immature stage (nymphs), there were five pools of three nymphs.

### DNA Extraction

Before DNA extraction, ticks were cleaned three times with 70% ethanol, 10% sodium hypochlorite, and sterile distilled water. DNA extraction was conducted using a DNeasy Blood and Tissues Kit (Qiagen) according to the manufacturer's protocol. The DNA products were stored at −20°C until use as templates for the PCR assay.

### Molecular Analysis

All extracted DNA samples were used as templates for PCR assays with specific bacterial primers for detecting the presence of *Coxiella, Rickettsia, Francisella*, and *Borrelia*. *Coxiella*-positive bacteria were identified through the *16S* rRNA, *groEL* (60-kDa chaperone heat shock protein B), and *rpoB* genes (β subunit of bacterial RNA polymerase). *Rickettsia* species were screened by PCR, targeting the *17-kDa* antigen, citrate synthase (*gltA*), outer membrane protein A (*ompA*), outer membrane protein B (*ompB*), and cell surface antigen (*sca4*) genes. PCR primer pairs for the detection of bacterial species, primer name, target genes, and size of the amplicons (bp) are shown in Table 1. The PCR product of the expected size from each corresponding primer pair (gene) was cloned and sequenced as a positive control. A sterile distilled water negative control was also included.

**Table 1:** Primers for PCR amplification used in this study.

**Organism**	**Target gene**	**Primer name**	**Sequence (5^**′**^-3^**′**^)**	**References**
Tick (Acari)	mt *16S* rDNA	16S+1	CTGCTCAATGATTTTTTAAATTGCTGTGG	(28)
		16S-1	CCGGTCTGAACTCAGATCAAGT	
*Rickettsia*	*17-kDa* antigen	RR17.61p	CATTGTTCGTCAGGTTGGCG	(29)
		RR17.492n	GCTCTTGCAACTTCTATGTT	
	*gltA*	RpCS.887p	GGGGGCCTGCTCACGGCGG	(30)
		RpCS.1258n	ATTGCAAAAAGTACAGTGAACA	
	*ompA*	RR190.70p	ATGGCGAATATTTCTCCAAAA	(31)
		RR190.602n	AGTGCAGCATTCGCTCCCCCT	
	*ompB*	RIC-F	CACCCAGCAAGGTAATAAGTTTA	(32)
		RIC-R	GCTATACCGCCTGTAGTAACAG	
	*sca4*	RrD749F	TGGTAGCATTAAAAGCTGATGG	(33)
		RrD1826R	TCTAAATKCTGCTGMATCAAT	
*Coxiella*	*16S* rRNA	COX-F	GGGGAAGAAAGTCTCAAGGGTAA	(34)
		COX-R	TGCATCGAATTAAACCACATGCT	
	*groEL*	CoxGrF1	TTTGAAAAYATGGGCGCKCAAATGGT	(35)
		CoxGrR2	CGRTCRCCAAARCCAGGTGC	
		CoxGrF2	GAAGTGGCTTCGCRTACWTCAGACG	
		CoxGrR1	CCAAARCCAGGTGCTTTYAC	
	*rpoB*	CoxrpoBF2	GGGCGNCAYGGWAAYAAAGGSGT	(35)
		CoxrpoBR1	CACCRAAHCGTTGACCRCCAAATTG	
		CoxrpoBF3	TCGAAGAYATGCCYTATTTAGAAG	
		CoxrpoBR3	AGCTTTMCCACCSARGGGTTGCTG	
*Borrelia*	*16S* rDNA	16SF1	ATAACGAAGAGTTTGATCCTGGC	(36)
		16SR	CAGCCGCACTTTCCAGTACG	
*Francisella*	*16S* rRNA	F11	TACCAGTTGGAAACGACTGT	(37)
		F5	CCTTTTTGAGTTTCGCTCC	

### DNA Purification, Sequencing, and Phylogenetic Analysis

The positive PCR products were purified with a Nucleospin Gel and PCR Clean-up Kit (Düren, Germany) and sequenced. The DNA sequence alignment of representative positive samples generated by this study was created using the CLUSTALW program. The nucleotide sequences were analyzed and blasted with the National Center for Biotechnology Information BLASTn database. Afterward, phylogenetic analyses were carried out using the maximum likelihood (ML) (38) and neighbor-joining (NJ) (39) methods (evaluated by bootstrap analysis with 1,000 replicates) for *Coxiella* and *Rickettsia* spp., respectively.

## Results

### Tick Collection

A total of 94 ticks were collected and identified. The species, number, and life stage of the ticks are shown in Table 2. The ticks belonged to two genera, *Haemaphysalis* and *Amblyomma*. Of the adult ticks, 13 males and 12 females belonged to *H. lagrangei*. The remaining ticks belonged to *A. testudinarium* (two female ticks). In the immature stage, *Haemaphysalis* sp. (22 nymphs and 28 larvae) was the most commonly collected, followed by *Amblyomma* sp. (17 nymphs). The species of all ticks included in the phylogenetic trees were confirmed by molecular methods, and their sequences were submitted to GenBank with the accession numbers shown in Tables 3, 4.

**Table 2:** Species, number, life stage of ticks, and results of bacterial infection in ticks analyzed by PCR (positive result of each bacterium) collected from vegetation in Chaiyaphum Province, Thailand.

**Tick species**	**Number of collected ticks**				**No. of PCR positive/No. of ticks**	
	**Male**	**Female**	**Nymph**	**Larva**	***Coxiella***	***Rickettsia***
*H. lagrangei*	13	12	0	0	16/25	1/25
*Haemaphysalis* sp.	0	0	22	28	3/3 individual, 4/7 pool	0/3 individual, 0/7 pool
*A. testudinarium*	0	2	0	0	2/2	1/2
*Amblyomma* sp.	0	0	17	0	2/2 individual, 5/5 pool	2/2 individual, 4/5 pool
Total	13	14	39	28	32/44	8/44

**Table 3:** Details of GenBank accession numbers of the *Coxiella* gene sequences and BLAST analysis of these sequences from tick samples collected from vegetation in Chaiyaphum Province, Thailand.

**Tick species and stage**	**Code (accession number of tick *16S* mt rDNA)**	**Percent identity (matching nucleotides/total) with closest** ***Coxiella*** **spp. sequences for each gene**		
		***16S* rRNA**	***groEL***	***rpoB***
*Amblyomma testudinarium*	PK33 (MZ490780)	99.6% (484/486) *Coxiella* sp. S027 (LT009437)	91.5% (529/578) *Coxiella* endosymbiont of *Ixodes hexagonus* Ihexa 1 (KP985500)	99% (486/491) *Coxiella* sp. S027 (LT174617)
*Amblyomma testudinarium*	PK48 (MZ490781)	99.8% (470/471) *Coxiella* sp. S027 (LT009437)	91% (523/575) *Coxiella* sp. CoxAscalpt 1 (MT000763)	99% (486/491) *Coxiella* sp. S027 (LT174617)
*Amblyomma testudinarium* nymph	PK138-140 (MZ490788)	99.8% (470/471) *Coxiella* sp. S027 (LT009437)	91% (523/575) *Coxiella* sp. CoxAscalpt 1 (MT000763)	99% (486/491) *Coxiella* sp. S027 (LT174617)
*Amblyomma testudinarium* nymph	PK51 (MZ490782)	99.6% (484/486) *Coxiella* sp. S027 (LT009437)	91.7% (530/578) *Coxiella* endosymbiont of *Ixodes hexagonus* Ihexa 1 (KP985500)	99% (486/491) *Coxiella* sp. S027 (LT174617)
*Amblyomma testudinarium* nymph	PK67-69 (MZ490783)	99.8% (483/484) *Coxiella* sp. S027 (LT009437)	91.7% (529/577) *Coxiella* endosymbiont of *Ixodes hexagonus* Ihexa 1 (KP985500)	99% (486/491) *Coxiella* sp. S027 (LT174617)
*Amblyomma testudinarium* nymph	PK81-83 (MZ490784), PK100-102 (MZ490785)	99.8% (485/486) *Coxiella* sp. S027 (LT009437)	91.7% (530/578) *Coxiella* endosymbiont of *Ixodes hexagonus* Ihexa 1 (KP985500)	99% (486/491) *Coxiella* sp. S027 (LT174617)
*Amblyomma testudinarium* nymph	PK121-123 (MZ490787)	99.6% (484/486) *Coxiella* sp. S027 (LT009437)	91.7% (530/578) *Coxiella* endosymbiont of *Ixodes hexagonus* Ihexa 1 (KP985500)	99% (486/491) *Coxiella* sp. S027 (LT174617)
*Amblyomma testudinarium* nymph	PK168 (MZ490790)	99.8% (485/486) *Coxiella* sp. S027 (LT009437)	91.2% (527/578) *Coxiella* endosymbiont of *Ixodes hexagonus* Ihexa 1 (KP985500)	99% (486/491) *Coxiella* sp. S027 (LT174617)
*Haemaphysalis lagrangei*	PK16 (MZ490778), PK17 (MZ490779)	99.8% (485/486) *Coxiella* sp. HLSD3 (JQ764626)	88.2% (516/585) *Coxiella* sp. TRG32 (MG874471)	94.3% 462/490 *Coxiella* sp. S002 (LT174612)
*Haemaphysalis lagrangei* nymph	PK146-150 (MZ490789)	99.8% (485/486) *Coxiella* sp. HLSD3 (JQ764626)	88.2% (516/585) *Coxiella* sp. TRG32 (MG874471)	94.3% 462/490 *Coxiella* sp. S002 (LT174612)
*Haemaphysalis obesa* larva	PK104-118 (MZ490786)	100% (486/486) *Coxiella* sp. TPSD8 (KC170759)	87.7% (514/585) *Coxiella* sp. TRG32 (MG874471)	92.2% 452/490 *Coxiella* sp. S002 (LT174612)

**Table 4:** Details of GenBank accession numbers of the *Rickettsia* gene sequences and BLAST analysis of these sequences from tick samples collected from vegetation in Chaiyaphum Province, Thailand.

**Tick species and stage**	**Code (accession number of tick *16s* mt rDNA)**	**Percent identity (matching nucleotides/total) with closet** ***Rickettsia*** **spp. sequences for each gene**				
		***17-kDa* antigen**	***gltA***	***ompA***	***ompB***	***sca4***
*Amblyomma testudinarium*	PK48 (MZ490781)	100% (411/411) *R. tamurae* Ate_1347 (LC379452), HM1 (AB812550), *Rickettsia* sp. 216 (KR733071); 100% (403/403) *R. tamurae* AT-1 (AB114825)	99.7% (368/369) *R. tamurae* AT-1 (AF394896)	100% (504/504) *R. tamurae* 1994_ISE6 (LC388793), AT-1 (DQ103259)	96.4% (758/786) *R. tamurae* AT-1 (DQ113910)	99.2% (1,003/1,011) *R. tamurae* AT-1 (DQ113911)
*Amblyomma testudinarium* nymph	PK51 (MZ490782), PK67-69 (MZ490783), PK81-83 (MZ490784), PK138-140 (MZ490788)	100% (411/411) *R. tamurae* Ate_1347 (LC379452), HM1 (AB812550), *Rickettsia* sp. 216 (KR733071); 100% (403/403) *R. tamurae* AT-1 (AB114825)	99.7% (368/369) *R. tamurae* AT-1 (AF394896)	100% (504/504) *R. tamurae* 1994_ISE6 (LC388793), AT-1 (DQ103259)	96.4% (758/786) *R. tamurae* AT-1 (DQ113910)	99.2% (1,003/1,011) *R. tamurae* AT-1 (DQ113911) (except PK138-140)
*Amblyomma testudinarium* nymph	PK168 (MZ490790)	100% (411/411) *R. tamurae* Ate_1347 (LC379452), HM1 (AB812550), *Rickettsia* sp. 216 (KR733071); 100% (403/403) *R. tamurae* AT-1 (AB114825)	99.7% (356/357) *R. tamurae* AT-1 (AF394896), HM1 (AB812551)	100% (504/504) *R. tamurae* 1994_ISE6 (LC388793), AT-1 (DQ103259)	96.2% (708/736) *R. tamurae* AT-1 (DQ113910)	*-* (faint band)
*Amblyomma testudinarium* nymph	PK100-102 (MZ490785)	100% (394/394) *Rickettsia* sp. ATT (AF483196)	100% (365/365) *Rickettsia* sp. 239 (KT753272)	100% (503/503) *R. tamurae* 1994_ISE6 (LC388793), AT-1 (DQ103259)	96% (752/783) *R. rhipicephali* 3-7-female 6-CWPP (CP003342)	97.3% (876/900) *Candidatus* Rickettsia thierseensis (MT424980), 97.3% (873/897) *R. fournieri* AUS118 (KF666473)
*Haemaphysalis lagrangei*	PK16 (MZ490778)	99.5% (409/411) *Rickettsia* sp. 315 (KT753267)	99.7% (320/238) *R. tamurae* Wuwei-Ha-1 (MH932020)	*-* (faint band)	*-* (faint band)	

### Detection of Bacteria

*Coxiella* and *Rickettsia* bacteria were detected in two genera of ticks (*Haemaphysalis* and *Amblyomma*), whereas *Francisella* sp. and *Borrelia* sp. were not identified in this study (Table 2). Single infection with *Coxiella* was detected in 16 of 25 *H. lagrangei* adult ticks (six of 13 males and 10 of 12 females), and single infection with *Rickettsia* was detected in one of 25 *H. lagrangei* females. Adult *A. testudinarium* ticks were infected with *Coxiella* (two of two females) and *Rickettsia* (one of two females). Single infection with *Coxiella* was detected in three of three nymphs and four of seven pools of *Haemaphysalis* ticks (one pool of four nymphs, two pools of five nymphs, and one pool of 15 larvae). In addition, two of two nymphs and five of five pools of *Amblyomma* ticks were positive for *Coxiella* and two of two nymphs and four of five pools were positive for *Rickettsia*. However, infection with *Rickettsia* was not detected in the immature stage of *Haemaphysalis*. Moreover, coinfection with these two bacteria was detected in infected adults of *H. lagrangei*. In addition, adults of *A. testudinarium* were coinfected with *Coxiella* and *Rickettsia*. Coinfection with these two bacteria was also present in *Amblyomma* nymphal ticks.

### DNA Sequencing and Phylogenetic Analysis

**Figure 1:**
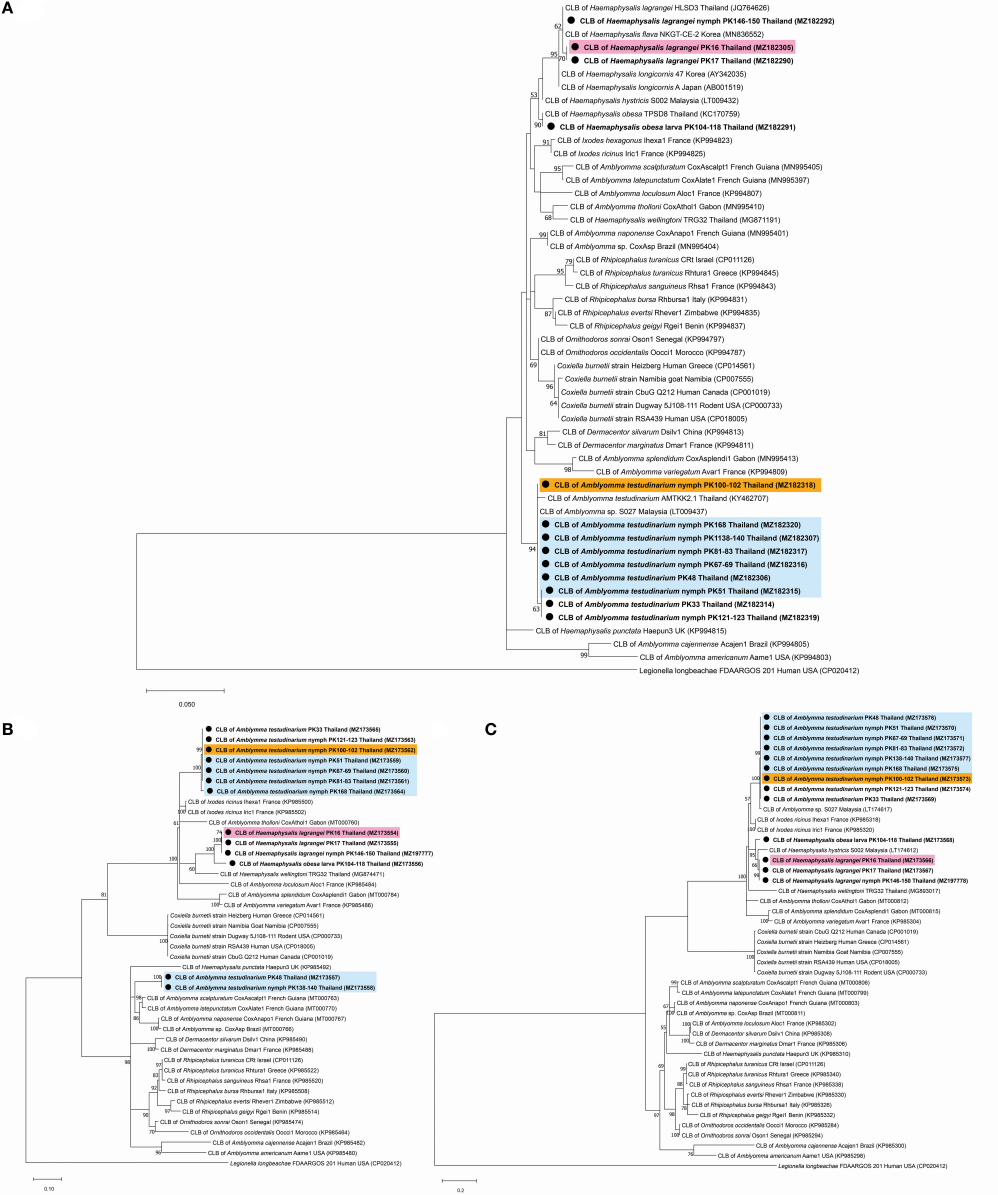
Phylogenetic tree of *Coxiella* species gene sequences using the maximum likelihood method with 1,000 bootstrap replicates (bootstrap values <50% are not shown). **(A)** *16S* rRNA gene: *Legionella longbeachae* was used as the outgroup. **(B)** *groEL* gene: *Legionella longbeachae* was used as the outgroup. **(C)** *rpoB* gene: *Legionella longbeachae* was used as the outgroup. DNA from *Coxiella* spp. amplified from ticks identified in this study is indicated by black dots and bold font. The scale bar indicates nucleotide substitutions per site.

A phylogenetic tree based on the partial sequences of *16S* rRNA revealed that all *Coxiella* sequences detected in this study belonged to two endosymbiotic groups (Figure 1A). *Coxiella* sequences detected in *H. lagrangei* and *Haemaphysalis obesa* were in the first group. PK16, PK17, and PK146-150 clustered with CLB of HLSD3 found in *H. lagrangei*, whereas PK104-118 grouped with CLB of TPSD8 detected in *H. obesa*. These sequences were closely related to those of CLB in *H. hystricis* S002 (LT009432), *H. longicornis* 47 (AY342035), and *H. longicornis* A (AB001519). *Coxiella* sequences in *A. testudinarium* and *Amblyomma* sp. formed a monophyletic clade and clustered together with CLB found in *Amblyomma* sp. S027 and *A. testudinarium* AMTKK2.1 from Malaysia and Thailand, respectively (Figure 1A). BLAST analysis of the *Coxiella 16S* rRNA and *groEL* and *rpoB* gene sequences from *Haemaphysalis* and *Amblyomma* ticks is shown in Table 3.

Interestingly, the BLAST results of the *groEL* gene sequences from this work showed DNA sequence identities of <92% compared to the existing sequences. Phylogenetic trees based on the partial sequences of *groEL* revealed that CLB in this study were clustered into three groups (Figure 1B). All sequences of *Coxiella* spp. in *Amblyomma* (except PK48 and PK138-140) grouped together as sister clades to the CLB of *I. ricinus*. CLB in *Haemaphysalis* grouped as sister clades with CLB detected in *H. wellingtoni* TRG32 (MG874471). Interestingly, the *groEL* sequences of PK48 and PK138-140 clearly formed separate clades from the other *Coxiella* spp. in *A. testudinarium* obtained in the first group, although they still formed a sister clade with the *Amblyomma* genus from other geographical regions. CLB in *Haemaphysalis* (PK16, PK17, PK104-118, and PK146-150) from this study (Figure 1B) formed a distinct clade from those of CLB in *Amblyomma*. Importantly, the BLAST results of the *rpoB* gene sequences from this work exhibited DNA sequence identities <95% compared to the existing sequences for *Haemaphysalis* (PK16, PK17, PK146-150, and PK104-118). Phylogenetic trees based on the partial sequences of *rpoB* revealed that all CLB sequences detected in this study were also divided into two groups. The first group included *Coxiella* spp. detected in *Amblyomma* ticks that formed a monophyletic clade and grouped together with CLB in *Amblyomma* sp. S027 (LT174617) from Malaysia. The second group included *Coxiella* sequences found in *Haemaphysalis* that formed an independent clade and grouped with CLB in *H. hystricis* S002 (LT174612) from Malaysia (Figure 1C).

**Figure 2:**
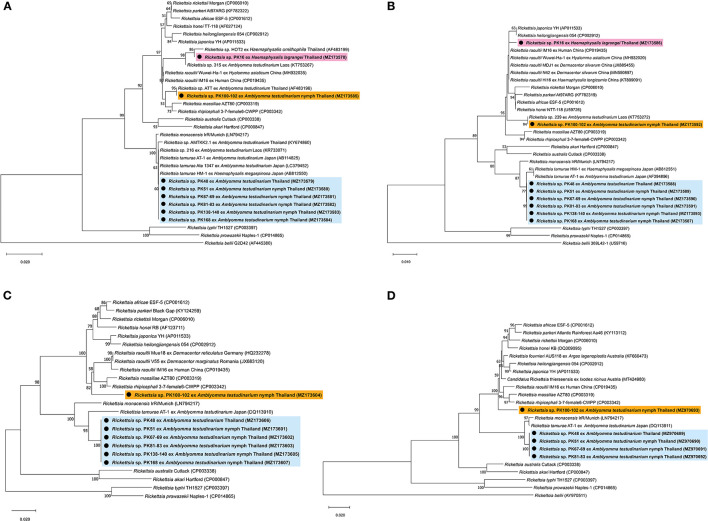
Phylogenetic tree of *Rickettsia* species using the neighbor-joining method with 1,000 bootstrap replicates (bootstrap values <50% are not shown). **(A)** *17-kDa* antigen gene: *Rickettsia bellii* was used as the outgroup. **(B)** *gltA* gene: *Rickettsia bellii* was used as the outgroup. **(C)** *ompB* gene: *Rickettsia typhi* and *Rickettsia prowazekii* were used as outgroups. **(D)** *sca4* gene: *Rickettsia bellii* was used as the outgroup. DNA from *Rickettsia* spp. amplified from ticks identified in this study is indicated by black dots and bold font. The scale bar indicates nucleotide substitutions per site.

BLAST analysis of the *Rickettsia 17-kDa* antigen, *gltA, ompA, ompB*, and *sca4* gene sequences from *Amblyomma* and *Haemaphysalis* ticks is shown in Table 4. The bands for *ompA* and *ompB* from PK16 were faint and could not be sequenced. Interestingly, the BLAST search results of all *ompB* genes studied herein showed <97% DNA sequence similarity compared to the existing *ompB* genes in *Rickettsia* spp. In addition, the BLAST search results of PK100-102 showed that the *Rickettsia ompB* gene sequences from *A. testudinarium* nymphal ticks had 96% sequence similarity to *Rickettsia rhipicephali* 3-7-female 6-CWPP (CP003342). Moreover, the BLAST search results of the *sca4* gene of PK100-102 showed 97.3% identity to *Candidatus* Rickettsia thierseensis (MT424980) and *Rickettsia fournieri* AUS118 (KF666473). Phylogenetic trees based on the partial sequences of the *17-kDa, gltA, ompB*, and *sca4* genes from *Rickettsia* sp. are shown in Figures 2A–D. Phylogenetic trees based on the partial sequences of the *17-kDa* (Figure 2A) and *gltA* (Figure 2B) genes indicated that the *Rickettsia* spp. detected in this study formed three groups. The first group (PK16) was clustered with *Rickettsia* sp. HOT2 and *Rickettsia* sp. 315 (*17-kDa* gene), and this group was within the *Rickettsia massiliae* subgroup of *Rickettsia*. Phylogenetically, the *gltA* PK16 sequence grouped with the *Rickettsia raoultii* clade (*R. massiliae* subgroup) and was closely related to the clades containing *Rickettsia japonica* and *Rickettsia heilongjiangensis*. The second group included PK100-102, which clustered together with *Rickettsia* sp. in *A. testudinarium* from Laos. The third group (PK48, PK51, PK67-69, PK81-83, PK138-140, and PK168) formed a sister clade with *R. tamurae* based on their *17-kDa* and *gltA* gene sequences.

However, phylogenetic analysis based on the partial sequence of the *ompA* gene was not included in this study because the *ompA* amplicons might be the products of contaminated reactions. In addition, phylogenetic trees based on the partial sequences of the *ompB* gene revealed that *Rickettsia* spp. formed two groups: the first group (including PK100-102) formed a sister clade to *R. raoultii, R. rhipicephali*, and *R. massiliae* (*R. massiliae* subgroup), and the second group (including PK48, PK51, PK67-69, PK81-83, PK138-140, and PK168) grouped within the clade containing *R. tamurae* (*Rickettsia helvetica* subgroup) (Figure 2C). A phylogenetic tree of the *sca4* gene (not including sequence PK138-140) showed the presence of two groups within SFG *Rickettsia* (Figure 2D), similar to the results for the *ompB* gene.

## Discussion

CLB have been identified in several tick genera, including *Haemaphysalis* and *Amblyomma*, and in at least two-thirds of tick species (15, 40, 41). Moreover, the tissue distribution of this symbiont within ticks showed that CLB specifically colonized the ovaries of female *Amblyomma cajennense* (42) and *H. longicornis* ticks (43), which also indicated that CLB is associated with the regulation of tick reproductive fitness (42, 44, 45). By using specific fluorescent foci, CLB were also observed in several tick tissues, including Malpighian tubules, salivary glands, and the midgut (46). In Thailand, the presence of CLB in the *Haemaphysalis* genus has also been documented (13, 24, 25). Our results added information on CLB in *H. lagrangei, H. obesa*, and *A. testudinarium* ticks from Chaiyaphum Province.

The phylogenetic tree based on ML analysis using the *16S* rRNA and *rpoB* genes showed that the detected CLB from both *Haemaphysalis* and *Amblyomma* ticks obtained in this study were clustered in the same clade as CLB gene sequences from similar genera. However, some *groEL* gene sequences of CLB in *A. testudinarium* ticks (accession numbers MZ173557 and MZ173558) detected in this study formed two separate clades. The *groEL* gene sequence of CLB in *A. testudinarium* (accession numbers MZ173557 and MZ173558) clustered with *Amblyomma* from other countries. Another group clustered with the *Ixodes* genus. This clade of *groEL* genes was also close to CLB associated with *H. wellingtoni* collected from domestic fowl from Trang Province, Thailand (25). The reason that the infection pattern shown by the *groEL* gene of CLB found in *A. testudinarium* was grouped with other previously published *Coxiella* sequences from the other tick genera may be horizontal gene transfer. A few examples have shown that accidental horizontal transmission occurs among host individuals, including during cofeeding. For example, the highly efficient exchange of the *rompA* gene of *Rickettsia conorii israelensis* was demonstrated between infected and uninfected *Rhipicephalus sanguineus* ticks feeding nearby each other on a dog that was not formerly infected with these bacteria (47). Interestingly, the results for the *16S* rRNA and *rpoB* markers in this study revealed that the CLB in *A. testudinarium, H. lagrangei*, and *H. obesa* ticks clustered together with known isolates, in contrast to the results found with the *groEL* marker. On the basis of the *groEL* phylogenetic analysis and BLAST results in this work, we found three groups of CLB: (1) CLB from *A. testudinarium* grouped as a sister clade with CLB from *I. ricinus*; (2) CLB from *H. lagrangei* was distantly related to CLB from *H. wellingtoni*; and (3) CLB from *A. testudinarium* grouped as a sister clade with CLB from *Amblyomma* ticks from French Guiana and Brazil.

*Rickettsia* spp. detected in ticks from this study grouped with SFG *Rickettsiae*, which are pathogenic bacteria. On the basis of the phylogenetic analysis, we showed that *Rickettsia* sp. detected from *H. lagrangei* tick (PK16) obtained in this study were clustered in different clades containing the rickettsial genes of *Amblyomma* ticks. Moreover, *Rickettsia* sp. detected from the *H. lagrangei* tick (PK16-MZ490778) grouped with *Rickettsia* sp. HOT2, which clustered with the *R. raoultii* clade based on the *17-kDa* gene. It has been reported that the presence of HOT2 *Rickettsia* has been detected in *Haemaphysalis ornithophila* ticks from Khao Yai National Park, Thailand (48). On the basis of the *gltA* gene sequence, PK16 grouped within the *R. raoultii* clade (*R. massiliae* subgroup). However, the *ompA* and *ompB* genes of PK16 could not be sequenced due to the presence of faint bands.

The BLAST analysis showed that the *ompA* genes of *Rickettsia* spp. collected from *Amblyomma* ticks were identical (100% DNA sequence identity) to those from *R. tamurae* AT-1 in all *Rickettsia* samples detected in this study. In this scenario, it is possible that the detection of the *ompA* sequences may have arisen due to PCR bias, resulting in this very surprising finding. An alternative explanation could be that the *ompA* amplicons were the product of a contaminated reaction. We did not include the phylogenetic analysis of the *ompA* gene in this study.

On the basis of the *gltA, ompB*, and *sca4* phylogenetic analyses and BLAST results from this work, we found two groups of SFG *Rickettsiae*: (1) SFG *Rickettsiae* that grouped as a sister clade with *R. tamurae* AT-1 (belonging to the *R. helvetica* subgroup) in *A. testudinarium* and (2) SFG *Rickettsiae* that was distantly related to *R. rhipicephali* 3-7-female 6-CWPP (belonging to the *R. massiliae* subgroup) in *A. testudinarium*. The pathogenic roles of these bacteria need to be studied further.

## Conclusions

From the results of the *groEL* phylogenetic analysis, CLB clades were found to group as a sister clades to CLB from *I. ricinus*, CLB from *H. wellingtoni*, and CLB from *Amblyomma* ticks from French Guiana and Brazil. In addition, on the basis of the *gltA, ompB*, and *sca4* phylogenetic analyses, SFG *Rickettsiae* formed two groups: a sister clade to *R. tamurae* AT-1 (belonging to the *R. helvetica* subgroup) and a clade distantly related to *R. rhipicephali* 3-7-female 6-CWPP (belonging to the *R. massiliae* subgroup). This study demonstrates the diversity of CLB and *Rickettsia* bacteria with their host ticks, which may act as potential vectors.

## Data Availability Statement

The datasets presented in this study can be found in online repositories. The names of the repository/repositories and accession number(s) can be found in the article/supplementary material.

## Author Contributions

AA wrote and edited manuscript and planned and designed the research with advice from VB and WT. PU wrote the original draft. VB and WT edited the manuscript. WK analyzed the data of the phylogenetic tree and writing. RS took care of the experiments. All authors contributed to the article and approved the submitted version.

## Funding

This research was supported by grants from Mahidol University, the Center of Excellence on Biodiversity (BDC) and the Office of Higher Education Commission (BDC-PG3-163005). This research was also supported by the Thailand Research Fund through the Royal Golden Jubilee PhD Program (Grant No. PHD 0175/2560) to PU.

## Conflict of Interest

The authors declare that the research was conducted in the absence of any commercial or financial relationships that could be construed as a potential conflict of interest.

## Publisher's Note

All claims expressed in this article are solely those of the authors and do not necessarily represent those of their affiliated organizations, or those of the publisher, the editors and the reviewers. Any product that may be evaluated in this article, or claim that may be made by its manufacturer, is not guaranteed or endorsed by the publisher.
